# Neuro-Empowerment of Executive Functions in the Workplace: The Reason Why

**DOI:** 10.3389/fpsyg.2020.01519

**Published:** 2020-07-31

**Authors:** Michela Balconi, Laura Angioletti, Davide Crivelli

**Affiliations:** ^1^Research Unit in Affective and Social Neuroscience, Catholic University of the Sacred Heart, Milan, Italy; ^2^Department of Psychology, Catholic University of the Sacred Heart, Milan, Italy

**Keywords:** neuromanagement, neurocognitive empowerment, executive functions, self-regulation, stress management, workplace

## Executive Functions Within the Company: a new discovery?

The successful achievement of pre-established working goals and the ability to respond appropriately to workplace demands depends both on efficient and flexible cognitive and social functioning.

Previous research has proposed that executive functions (EFs) play an essential role in work performance, with successful professionals displaying better social, cognitive, and executive functioning (Bailey, 2007; Willoughby and Blair, 2016). Therefore, the demand of assessment procedures and empowerment protocols dedicated to the EFs is growing rapidly.

EFs are considered a family of top-down mental processes including inhibition (self-control and interference control), working memory, and cognitive flexibility (Miyake et al., 2000; Diamond, 2013). They are high-level cognitive functions that foster goal-directed behavior and are a pre-requisite for sustained focusing, regulation of attention resources and automatic responses, and rapid and flexible adjustment to the changeable requests of the environment (Miller and Cohen, 2001; Burgess and Simons, 2005). These components sustain more complex cognitive functions—such as reasoning, planning, decision-making, creativity, and problem solving—which represent critical skills for professional success and optimal workplace performance. As posited by Diamond (2013), “*EFs make it possible for us to mentally play with ideas, quickly and flexibly adapt to changed circumstances, take time to consider what to do next, resist temptations, stay focused, and meet novel, unanticipated challenges*” (p. 155).

In particular, according to an integrated EFs and self-regulation model (Hofmann et al., 2012), working memory capacity, behavioral inhibition, and flexibility act as the fertile ground for fostering active representation of multiple self-regulatory goals, adaptively switching and orienting cognitive resources toward individual goals while actively inhibiting distracters, suppressing maladaptive habits and mindless behavior, and regulating unwanted affective reactions and dysfunctional distress responses. The ability to self-regulate—together with the ability to reflexively become aware of own communication, relational, and affective schemata and to interpret others' mental states—is then deeply linked to EFs.

Notably, it is today acknowledged that EFs might support social skills and emotion-regulation, which also play a crucial role for successful management of social dynamics, interpersonal relations, and adaptive stress-management (Cacioppo and Cacioppo, 2020). Building on such premises, we suggest that they together subserve the development of a domain-independent repertoire of soft skills—such as adaptive management of the stress load, empathy (intended as the ability to interpret and understand others' intentions, desires, and affective states), interpersonal and communication efficacy, and leadership—and then deserve peculiar attention with implications for both assessment and human resources (HR) development programs. If analyzed via neuroscientific models, they might provide important advantages for the enhancement of mental and relational resources: indeed, the environment where business activities grow and develop is dynamic, continuously changing, and increasingly complex. Professionals working in and with such context have to be flexible, prone to change and able to promptly adapt to novel situations, quick in finding creative solutions to problems, proficient in managing work-related stressors (Chandola et al., 2010), and effective in communicating and creating positive interpersonal relationship (Balconi et al., 2017b; Crivelli and Balconi, 2017). In a highly competitive business environment such as the one we are immersed in, organizations might achieve and maintain an advantage over competitors by investing on the identification and growth of valuable human resources (Obisi, 2011; Collings et al., 2019).

Yet, despite the relevance of EFs as the ground for complex cognitive and social processes and their potential role as precursors for self-regulation and other core professional soft skills, an overarching framework for exploring EFs at work is still absent in the neuromanagement field. For these reasons, this work aims at fuelling the debate on the relevance of EFs for professionals' cognitive, affective, and relational functioning by presenting a novel framework for the investigation and development of EFs at work, and at discussing, via applied examples, future challenges and opportunities for research and practice of an effective integration of neuroscientific models and methods with HR development.

## EFs in the Triadic Model for Assessment and Neuroempowerment

**Figure 1:**
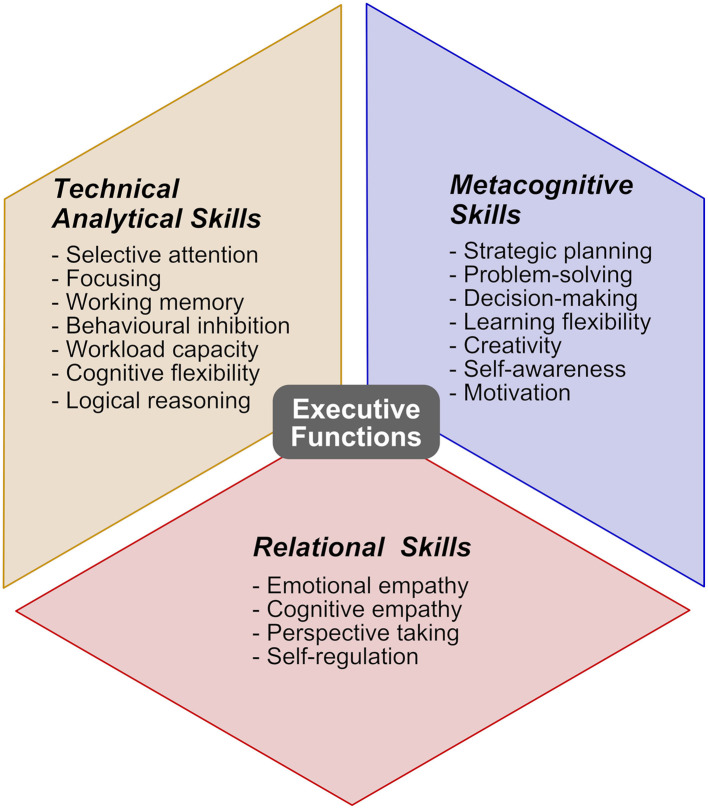
The triadic model of neuroempowerment for the executive functions at the workplace.

According to the triadic model for talent assessment and neuroempowerment devised by Balconi and collaborators (Figure 1), individual professional potential might be explored by taking into account three main clusters of competencies: (i) technical-analytical skills, (ii) metacognitive skills, and (iii) relational skills.

Technical skills are specific to the function and business area and depend on the person's educational and experiential background (Silzer and Church, 2009; Dessler, 2016). Typical examples are logical reasoning applied to specific problem-solving. Analytical skills, compared with technical ones, are characterized by a lower level of specificity with regard to organizational positions, in that they concern domain-independent skills that are required in transversal areas of applications, such as selective attention, focusing, working memory, and behavioral inhibition.

In contrast, metacognitive skills are higher-order cognitive process that, besides being necessary for autonomous everyday functioning, are peculiarly important as the complexity of the acting context increases, up to the highest levels of professional life. They include the definition of complex behavioral and cognitive strategies, awareness of own cognitive and affective processes, and the use of knowledge related to mental processes to monitor and control them, allowing the correct allocation of cognitive resources (Dunning et al., 2018). Metacognitive skills generally encompass strategic planning, decision-making under uncertainty, and learning flexibility; self-awareness (e.g., the proficiency in aptly appraising subjective strong and weak points); and ability to focus on intrinsic motivational drives.

Finally, relational skills, a core component in most of available models on the determinants of talent in organizations (Dries and Pepermans, 2007; Silzer and Church, 2009), are in the triadic model represented by individual ability in understanding and monitoring own and others' affective states (emotional empathy), as well as others' point of view, beliefs and intentions (cognitive empathy), by the efficiency in managing social interactions, and by the propensity toward interpersonal relations. As elements of the overarching construct of emotional intelligence, those abilities contribute to the success of a professional in social and organizational life (Dries and Pepermans, 2007).

On one hand, the top-down mental processes that constitute the core of human EFs—i.e., inhibition mechanisms, working memory and information-processing capacity, and cognitive flexibility (Miyake et al., 2000; Diamond, 2013) —transversely connote the internal structure of the triadic model and the interdependent set of skills that constitute its three components rather than being its unique cornerstones, consistent with latest integrated accounts of EFs and self-regulation (Hofmann et al., 2012). On the other hand, the model might act as a framework for devising and implementing novel integrated protocols for talent development and neurocognitive empowerment at the workplace or profiling of high-level competencies and hard/soft skills necessary for optimal job-related performance.

Between the others, the model already proved to be a useful map for designing both group-based interventions on stress management and neurocognitive efficiency dedicated to senior managerial positions (Crivelli et al., 2019b; Fronda et al., 2019) and personalized age-management interventions based on tailored neurocognitive empowerment protocols for higher EFs supported by wearable neurotechnologies.

## Empowering EFs: Applied Neurocognitive Protocols

In the field of neuromanagement there is the impellent need for effective and efficient protocols to empower higher EFs and self-regulation. Recently, we developed and tested an innovative training protocol mediated by wearable neurotechnologies, devised for the optimization of stress management, focusing, and executive control in stressful professional contexts, with people who occupy top management positions (Crivelli et al., 2019b). Top managers were selected based on their formal company position, with the specific role as manager being one who manages a group of resources of at least 10 people and has at least 5 years of experience in people management. They belonged to some main companies representing top national or international companies in specific sectors (services, transport, food, consulting, advertising, and insurance).

This innovative protocol specifically combined mindfulness practice and EF potentiation with the use of a wearable neurofeedback (NF) system managed via smartphone, and it has been validated by previous research in both experimental and applied contexts (Balconi et al., 2017a, 2019a,b; Crivelli et al., 2019a,c). NF devices collect electroencephalographic (EEG) brain waves signal and effectively provide real-time feedback on the person's mind–body state activity (Gruzelier, 2014). NF wearable devices reliability in quality signal was previously compared with EEG signal and demonstrated good quality standard and precise feedback (Bhayee et al., 2016; Balconi et al., 2017a). Compared to traditional NF, NF wearable device added value lies in the high usability, low cost, and portability.

The feasibility and efficacy of the empowerment protocol have been tested with 16 managers (Crivelli et al., 2019b). The training period lasted 2 weeks and was constituted by brief daily activities. The neuroempowerment protocol was based on breathing awareness practices derived from mindfulness practice, which were supported by a dedicated wearable NF device, namely, a non-invasive EEG recording system connected to a smartphone app that was devised to support mental practices and help foster self-awareness and self-regulation via real-time acoustic feedbacks on changes of the EEG signature of practicer's mindset.

At the end of the training, participants showed a significant decrease of perceived stress, anxiety, anger and mental fatigue, coupled with greater neurocognitive efficiency during challenging cognitive tasks, improved electrophysiological markers of relaxation and focusing, and improved autonomic markers of parasympathetic recovery when exposed to cognitive stressors. Such study was the first systematic investigation of a neurotechnology-mediated empowerment protocol in an organization and with top management professionals. Though highly innovative, the first experimentation was, however, primarily targeted on just two core skills: self-awareness and executive control of attention resources and stress response. The inclusion of NF wearable devices provides the opportunity to gather information on implicit processes that otherwise will remain at preconscious level, and difficult to identify. It can be considered a reliable scientific technique in applied neuroscientific protocols and the new challenges will concern the development of more varied software and training for the enhancement of executive functions.

Our latest applied research activities have then focused on the development of a revised empowerment protocol that delves its roots deep into the proposed triadic model for talent assessment and development. Such protocol was devised to make the practicer train integratedly the three core clusters of competencies and, in particular, to offer a personalized training opportunity for empowering of analytical, executive, metacognitive, and social skills in aging senior managers. The integrated training approach starts with a complete assessment of practicer's cognitive, executive and affective functioning and then the previously described NF training via wearable device is integrated into a training schedule with activities dedicated to both technical and analytical (cognitive flexibility, working memory, and reasoning), metacognitive (problem-solving, multitasking, and creativity), and relational skills (perspective-taking, social self-awareness, and self-regulation). The intensive protocol lasts 3 weeks and includes pre/post-training assessment based on psychometric, neuropsychological, and cognitive performance measures, as well as detection of neurometric and autonomic markers of neurocognitive efficiency and adaptive stress management.

Preliminary evidence on a first pilot case highlight greater neurocognitive efficiency featured by an increase in working memory performance, cognitive flexibility, problem-solving, inhibitory control, self-awareness and self-regulation, as well as a decrease in perceived stress levels both at behavioral and neurophysiological level. This work is configured as an evolution of the previous research protocol which strives to train and hold together all dimensions of the triadic model. Furthermore, it could be applied as a valid option for preventive age management interventions in high-level professional contexts.

## Conclusion

Neuromanagement was born from the need to understand mental processes subserving motives, attitudes, and behaviors of professionals in organizations with the final goal of predicting, modifying, and/or enhancing them (Becker et al., 2011; Balconi et al., 2017b; Murray and Antonakis, 2019). Yet, the application of neuroscience in organizational contexts still represents a frontier when we move to field applications. Several authors argue that the lack of familiarity with neuroscientific methods (Nofal et al., 2018), the uncertainty about expectations (Murray and Antonakis, 2019), the resistance compared to “looking in the Black Box” (Becker et al., 2011), and the presence of ethical issues have probably made the professionals of the organizations rather cautious (Butler et al., 2016).

Nonetheless, while the young field of neuromanagement is constantly evolving to adapt to changing professional challenges, the integration of social-cognitive neuroscience and organizational disciplines already seemed to offer a valuable help for both methodological-theoretical and technical reasons. Firstly, neuroscientific disciplines include at their core a primary interest for human mind and its higher cognitive and social functions, and their application strongly rely on the integration of multiple levels of analyses—from overt behavior and subjective experiences to covert central and peripheral physiological processes that accompany and support them. Secondly, the neuroscience approach typically implements methodologies and tools that proved to effectively tap on mental processes guiding self-regulation, social skills, and higher cognition even at the workplace. That might contribute to the effective design of HR management practices and policies, as well as to the identification and development of mission-critical professionals (Waldman et al., 2017).

Aware of ethical implications of neurocognitive enhancement on individuals and society but also of the benefits and criticalities of its application in professional contexts, EF neuroempowerment holds potential for the improvement of organizations effectiveness and productivity.

## Author Contributions

MB and DC contributed to the conception and design of the study. MB, DC, and LA wrote the first draft, contributed to manuscript revision, read, and approved the submitted version.

## Conflict of Interest

The authors declare that the research was conducted in the absence of any commercial or financial relationships that could be construed as a potential conflict of interest.
